# Landmarks and Surface Markings of the Human Body

**Published:** 1905-03

**Authors:** 


### Reviews of Books.

**Landmarks and Surface Markings of the Human Body.** By LOUIS BATHE RAWLING, M.B., B.C. Cantab., F.R.C.S. Pp. vi., 76. London: H. K. Lewis. 1904.

This book, as it supplies a want in the series of text-books, will be greatly appreciated not only by the student before examination, but by the busy practitioner and operator afterwards. It presents what in our opinion Holden's landmarks lacked, and that is illustrations and diagrams, and the illustrations are especially clear and explanatory, each picture being furnished with a list on the same page of the various structures depicted, distinctly indicated by a number. This arrangement, apart from the text, makes the work of considerable value to anyone who wishes to refresh his memory as to the exact geography of a part with which he has to deal, and who has not the time to consult larger works on anatomy.

A part of the book that we think will be found useful is the appendix, which gives the length of various mucous passages and other organs; but we missed what we first looked for, namely the average length of the alimentary canal from the incisors to the cardiac opening in the stomach. It would perhaps be the author's pleasure to add this in his second edition.

